# Deficient visuomotor hand coordination in normal pressure hydrocephalus

**DOI:** 10.1007/s00415-021-10445-5

**Published:** 2021-02-17

**Authors:** Hannah Köster, Katharina Müller-Schmitz, Aschwin G. J. Kolman, Rüdiger J. Seitz

**Affiliations:** 1 Department of Neurology, Medical Faculty, Centre for Neurology and Neuropsychiatry, LVR-Klinikum Düsseldorf, Heinrich-Heine-University Düsseldorf, Bergische Landstrasse 2, 40629 Düsseldorf, Germany; 2 Florey Neuroscience Institutes, Melbourne, VIC Australia

**Keywords:** Visuomotor coordination, Lumbar puncture, Tap-test, Supplementary motor area, Normal pressure hydrocephalus

## Abstract

**Objective:**

To investigate if visuomotor coordination of hand movements is impaired in patients with normal pressure hydrocephalus (NPH) identified by dedicated testing procedures.

**Methods:**

Forty-seven patients admitted for diagnostic workup for suspected NPH were studied prospectively with MRI, testing of cognitive and motor functions, lumbar puncture, and visuomotor coordination of hand movements using the PABLO^R^-device before and after a spinal tap of 40–50 ml CSF. Statistical analyses were carried out with repeated measures ANOVA and non-parametric correlation analyses.

**Results:**

Fourteen patients were found to suffer from ideopathic NPH. They were severely impaired in visuomotor control of intermittent arm movements in comparison to patients who were found not to be affected by NPH (*n* = 18). In the patients with NPH the deficient arm control was improved after the spinal tap in proportion to the improvement of gait. There was no improvement of cognitive and motor functions in the patients not affected by NPH, while the patients with possible NPH (*n* = 15) showed intermediate deficit and improvement patterns.

Interpretation: Our data underline the importance of a multiparametric assessment of NPH and provide evidence for a motor control deficit in idiopathic NPH involving leg and arm movements. It is suggested that this motor control deficit resulted from an affection of the output tracts from the supplementary motor area in the periventricular vicinity.

## Introduction

Normal-pressure hydrocephalus (NPH) established as a neurological syndrome in the pre-neuroimaging era [1] is characterized by cognitive disturbances, a broad based, shuffling gait and urinary incontinence in the presence of a communicating hydrocephalus and a normal opening pressure upon lumbar puncture [2]. In the elderly, aged 80 and higher, the estimated prevalence is approximately 9% and, thus, far more frequent than in seniors younger than 80 years [3]. Early shunting has been reported to be an effective treatment [4–6]. However, as the clinical appearance may vary among patients such that the typical triad is present in less than 60% of the patients [7, 8], the existence of idiopathic NPH has been questioned more recently [9–11]. With respect to this uncertainty we have adopted quantitative investigator-independent tests which have been validated in the literature to assess the neurological impairments and their putative improvement after the spinal tap [12].

In particular, we also applied a quantitative investigator-independent test to explore the hypothesis that NPH does not only affect urine continence and cognitive and gait functions but also functioning of the arm and hand. Specifically, we were interested to assess visuomotor hand coordination using quantitative measures of hand-arm functions that are performed in daily life. For this purpose, we studied hand movement control of items on a computer screen which is a common and widely used task in the present time. We will show that visuomotor coordination of manual actions is impaired in NPH suggesting that these patients suffer from a more general motor deficit than has been appreciated so far.

## Patients and methods

### Patient classification

Forty-seven patients of both sexes admitted for diagnostic work-up of putative NPH between August 2015 and June 2017 who had agreed to participate in this experimental investigation were prospectively included into this study. The patients suffered from a recently insidiously progressing impairment of gait and cognitive abilities as well as urine incontinence and have communicating internal hydrocephalus on cranial MRI. According to the International Guidelines the ventricular enlargement was associated with a patent Sylvian aqueduct and absence of a macroscopic obstruction of CSF flow, lack of cortical atrophy, presence of periventricular water content, and an increased callosal angel in the coronal plane [11, 13]. Patients with a prior severe head trauma, meningitis, intracranial hemorrhage or a previously diagnosed neurodegenerative disorder such as Parkinson’s disease or primary dementia were excluded. The patients were subjected to neuropsychological testing and a standardized testing of gait and mobility within 24 h before and 24–48 h after a spinal tap of 40–50 ml cerebrospinal fluid. The greatest change in performance has been described to be in the 24–48 time window after the spinal tap [13]. According to the results of these diagnostic procedures the patients were classified as typical NPH, possible NPH, and no NPH in accordance with the clinical classification as proposed by Relkin et al. [14].

### Clinical investigations

The neuropsychological tests comprised (I) the clock drawing test [15] to assess visual-spatial organization, (II) the German version of the verbal digit span and block tapping to assess auditory and visual memory span and working memory (WMS-R [16]), (III) the Syndrom Kurz Test (SKT; screening for memory and attention [17]), (IV) figure drawing and remembering (CERAD [18]), and (V) the mosaic test [19] to assess visuoconstruction, and alertness [intrinsic and phasic).

Gait and mobility were assessed using a repertoire of dedicated tests as detailed previously [12]. These tests included (I) the German version of the De Morton Mobility Index [20]), (II) 10 m Walking Test (10MWT [21]), and (III) Time-up-and-Go-Test (TUG [22]). The 10MWT and TUG were hand measured and the average of three attempts was used. Also, the patients were subjected to an identification test of seniors at risk before the spinal tap [23]. After the spinal tap, the patients were asked to give a global rating of the change they perceived being either worse, about the same or better for assessing the subjective satisfaction [24].

CSF samples were obtained by lumbar puncture after informed consent and processed as described elsewhere [25]. In short, quantitative analyses were performed by a commercial laboratory partner and classified according to standardized cut-off values (MVZ Synlab Leverkusen, Germany). Standardized sandwich ELISA methods were used for measurement of the core biomarkers, namely the INNOTEST^®^ -AMYLOID (1–42), INNOTEST^®^ hTAU Ag, INNOTEST^®^ PHOSPHO TAU (181P). Neuron-specific enolase and S100B were measured using the fully automated commercially available chemoluminescence immunoassays LIAISON^®^ S100 and LIAISON^®^ NSE (DiaSorin, Italy).

### Visuomotor hand coordination

Hand movements were assessed with the so-called PABLO^R^-device as detailed previously [26]. In short, the patients viewed a virtual landscape on a computer screen.

In the *balloon task* the scenario displayed mountains, houses, trees and clouds and a hot air balloon which moved with constant speed on screen from the left to the right for 2 min. The balloon had to be steered by a handle involving rotating hand movements. Clockwise hand movements made the balloon going up and anti-clockwise hand movements made the balloon go down on screen. The patients’ task was to steer the balloon such that it would not bump into a mountain or cloud. When the subject failed in performing this highly predictable task and the balloon bumped into the obstacles, the movement speed of the balloon was decreased for a few seconds. There were 10 levels of task difficulty given by an increasing travel speed of the balloon.

**Fig. 1:**
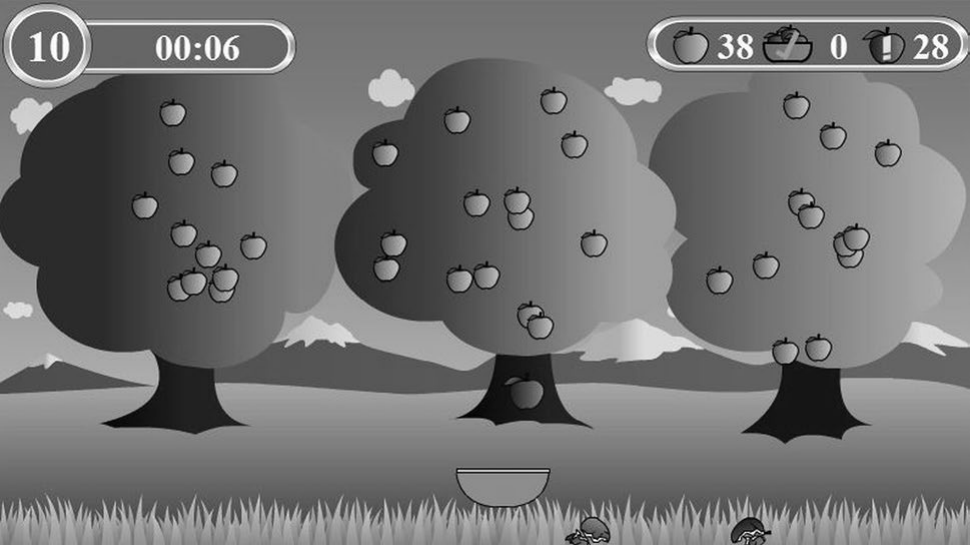
Screen view of the apple collecting task as provided in the PABLO^R^-device [26]. The falling apple can be seen in front of the stem of the middle tree with the basket for collection underneath. Two damaged apples can be seen on the floor

In the *collecting apple task,* there were three apple trees with green apples (Fig. 1). In random order one of the apples turned red and shortly thereafter fell down for the task duration of 2 min. On the floor, there was a basket which had to be positioned such that the apple would fall into it. The basket could be moved back and forth with the PABLO^R^-device using rotating movements of the hand holding the handle. Clockwise hand movements made the basket move left and anti-clockwise hand movements made the basket move right on the screen. The patients’ task was to collect the falling apples with the basket. Misses were indicated by damaged apples remaining on the floor. There were 10 levels of task difficulty given by an increased frequency and speed of the falling apples.

The study was approved by the Ethics Committee of the Medical Faculty of the Heinrich-Heine-University Düsseldorf (#5148).

### Statistical analysis

On the individual level of each patient an improvement of the neuropsychological performance and of the Demmi index after the tap-test was determined for each test as a categorical change in the age, gender and education related mean reference values in comparison to the pre-test values. Furthermore, an improvement in the 10MWT of > 10% was set as a cutoff for improvement (13). In addition, the data were entered into a spreadsheet of SPSS (IBM, Germany) for group statistics. The values of gait performance and of the PABLO^R^-device were entered as raw values. A repeated-measures ANOVA with post-hoc paired *t* tests of the changes was calculated. *T* values with alpha-corrections for multiple comparisons were calculated and converted to p-values. Significant change was defined to exceed *p* < 0.05. Also non-parametric correlation analyses using Spearman Rank correlations (SRC) were calculated. Significant SRC had to exceed a *p* < 0.05.

## Results

Each patient could perform the neuropsychological test battery and the gait assessment tests as summarized in Table 1. Fourteen patients were identified to belong to the NPH group. They presented the typical clinical symptoms of NPH including cognitive deficits, a slurred, unsteady and slowed gait, and reported urine incontinence. On tests of gait function and cognition they were impaired as compared to age-related norms with an increased risk of falling (Table 1). Also, these patients improved in cognitive abilities, gait parameters, and incontinence after the spinal tap as summarized in Table 1. Fifteen patients with possible NPH (posNPH) showed also a slowed gait but less consistent response to the tap test. They failed to improve concerning incontinence, neuropsychological testing and gait functions as evident from Table 1. Eighteen patients with a similarly slowed gait were found upon clinical examination and evaluation of their brain scans not to be compatible with NPH but rather to suffer from other conditions. Accordingly, they did not improve upon the spinal tap as shown in Table 1 (noNPH). The patients of this group turned out to suffer primarily from other neurological disorders such as cerebral microangiopathy, dementia of other origin, Parkinson’s disease, stenosis of the spinal canal, spasticity of undetermined cause, and polyneuropathy.

**Table 1:** Demographic Data of the Patients

Patient group	NPH	posNPH	no NPH
Number (*n*)	14	15	18
Females / Males	5/9	8/7	10/8
Age (years)	72 ± 6	74 ± 7	73 ± 8
Right handedness	12/2	15/0	17/1
Urine incontinence (*n*)	10	9	11
ISAR	1.7 ± 1.1	1.9 ± 1.1	1.4 ± 1.1
DEMMI	65.3 ± 17.6	65.1 ± 15.5	71.3 ± 9.5
Time-up-and-go test (s)^#^	16.6 ± 7.6 ^##^*	13.9 ± 5.9	11.9 ± 3.3
Walking speed (m/s)^###^	1.1 ± 0.5	1.1 ± 0.4	1.2 ± 0.4
Improvement of cognition after tap** (*n*)	10	5	5
Global perceived effect after tap (*n*)	9	3	3
ß-amyloid (> 500 pg/ml)	755 ± 229	739 ± 258	694 ± 369
tau protein (< 500 pg/ml)	231 ± 119	266 ± 142	303 ± 159
phospho-tau (< 61 pg/ml)	32 ± 15	36 ± 14	35 ± 16
neuron specific enolase (< 13 µg/l)	11 ± 6	13 ± 5	39 ± 82
S100 protein (< 2700 pg/ml)	2906 ± 633	3478 ± 1038	3567 ± 1779

Legend: mean ± standard deviation

*n* patients, *s* second

^*^Reduction of time after spinal tap (*p* < 0.02, corrected for multiple comparisons)

^**^Patients with change in neuropsychological tests, ISAR: Identification of seniors at risk test; > 2 pathological [23], DEMMI: De Morton mobility index, improvement: > 10 points [20]

^#^Age-related normal range: 8.2–10.2 s [38]

^##^Difference (p < 0.05) between NPH and no NPH

^###^Age-related walking speed: 2.1 ± 0.35 (SD) m/s [39]

The patients of each group were some seventy years of age and in the majority right-handed (Table 1). Incontinence was far more frequent in the patients with NPH and of the posNPH group. These patients had a slightly higher risk as indicated by the ISAR test than the patients with no NPH. The patients of all three groups were below the normal range concerning mobility and gait velocity. The patients with NPH were particularly impaired in the TUG (*p* < 0.05) but improved in this test after the spinal tap (*p* < 0.02). In addition, the majority of the patients with NPH improved in neuropsychological testing, while some improvement was found only in a small proportion of patients in the other two groups (Table 1). The subjective feeling of improvement was reflected by the global perceived effect test [25] in the patients with NPH, while this was less so in the other two groups. In the patients with NPH and in the patients with posNPH the S100 protein was elevated, while in the no NPH group both the S100 protein and neuron-specific enolase were markedly elevated (Table 1).

**Fig. 2:**
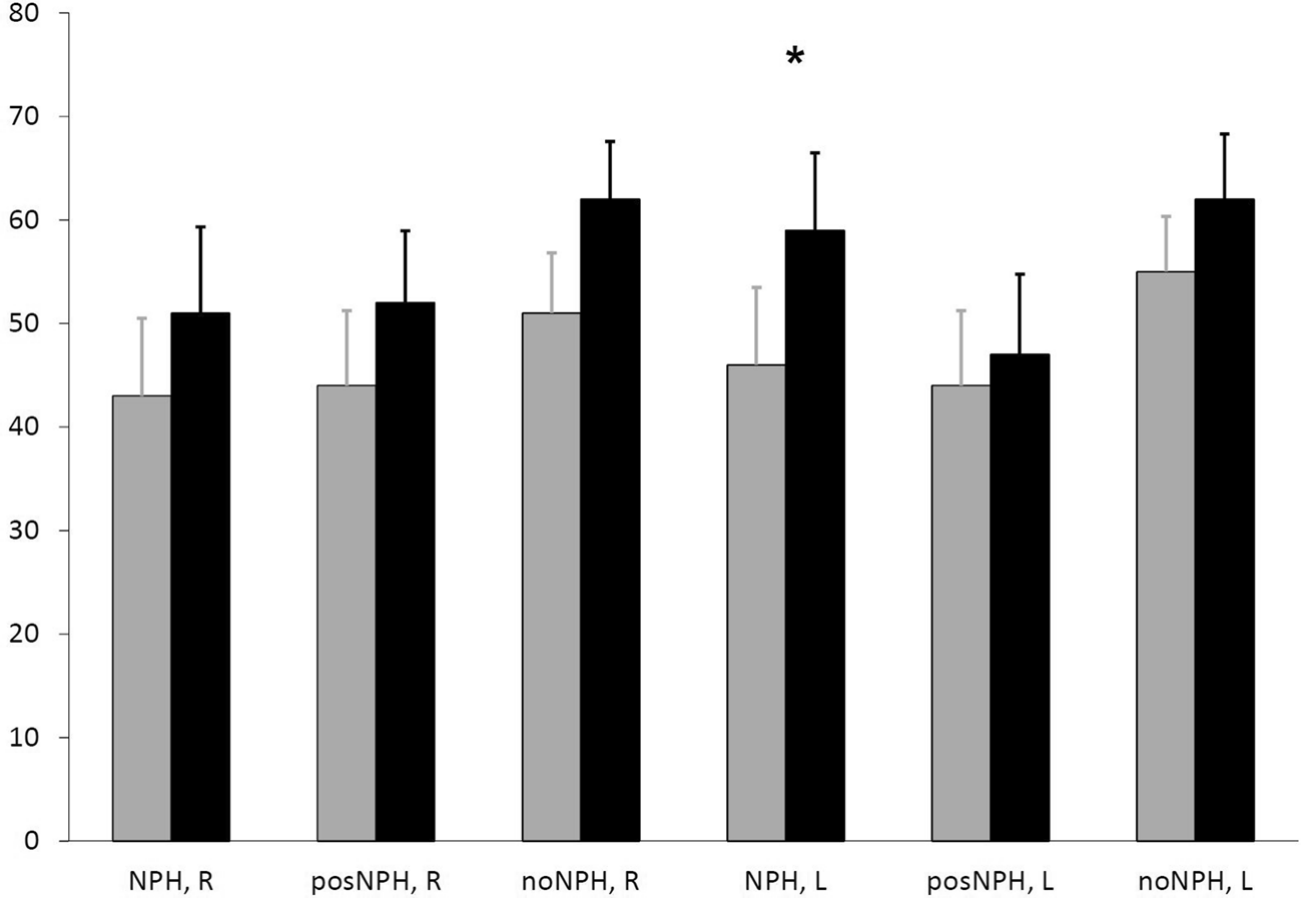
Improvement of the performance rate of the apple collecting task from before (grey columns) to after the spinal tap (black columns) for either hand in the three patient groups. Only improvement of the left hand in the patients with NPH was significantly (*p* < 0.02). *R* right hand, *L* left hand. Error bars: standard error

In the visuomotor hand coordination tasks, the patients with NPH displayed a differential pattern of impairment. Steering of the moving balloon was performed with a similar level of success by all patients in either patient group (Table 2). Right- and left-hand performance was virtually identical. Importantly, raising the level of task difficulty affected the performance rate slightly such that the patients bumped into the obstacles more frequently. But fast steering movements could be performed by all patients of each group. In contrast, in the apple collecting task the patients with NPH and posNPH were slightly impaired as compared to the noNPH patients with either hand (Table 2). The patients failed to collect apples with the basked at a higher task difficulty with either hand which resulted in an incomplete performance rate as can be seen in Table 2. After the spinal tap, the patients of the three groups were improved with the right hand. The patients with NPH improved also with their left hand which was significant, while there was virtually no improvement of the left hand in the patients with posNPH and no NPH (Fig. 2).

**Table 2:** Task performance in visuomotor coordination

Patient group	NPH	posNPH	No NPH
Balloon steering			
Right hand	88 ± 12	86 ± 16	94 ± 6
All levels	14/14	14/15	18/18
Left hand	94 ± 8	84 ± 22	93 ± 9
All levels	14/14	12/15	18/18
Collecting apples			
Right hand	43 ± 27	44 ± 27	51 ± 24
All levels	5/14	5/15	5/18
Left hand	46 ± 27*	44 ± 27	55 ± 22
All levels	4/14	4/15	9/18

Legend: points achieved by all patients at lowest difficulty (mean ± standard deviation), number of patients at the highest level of difficulty;

*Improvement after spinal tap (*p* < 0.02)

Correlation analysis revealed that after the spinal tap gait mobility correlated with the performance in the visuomotor hand coordination tasks in the three patient groups in a differentiated manner (Table 3). Task performance correlated positively with gait velocity as assessed with the 10MWT and inversely with the time needed to complete the TUG. Visuomotor coordination in the balloon task at a higher level of task difficulty correlated with gait velocity and performance in the TUG for both hands in the patients with NPH and in the patients with no NPH, while such correlations were found in the patients with posNPH only for the right hand. In the apple collecting task correlations of hand coordination with gait performance were found at the lowest level of performance for both hands only in the patients with NPH (Table 3).

**Table 3:** Relation of gait performance and visuomotor coordination

Patient group	NPH	posNPH	no NPH
Balloon steering			
Right hand			
10MWT	0.701 (0.003)	0.561 (0.023)	0.470 (0.025)
TUG	– 0.743 (0.001)	– 0.718 (0.003)	– 0.465 (0.026)
Left hand			
10MWT	0.619 (0.008)	n.s	0.609 (0.004)
TUG	– 0.859 (0.000)	n.s	– 0.533 (0.011)
Collecting apples			
Right hand			
10MWT	0.666 (0.005)	0.400 (0.070)	n.s
TUG	– 0.688 (0.003)	n.s	n.s
Left hand			
10MWT	0.481 (0.041)	n.s	0.412 (0.045)
TUG	– 0.578 (0.015)	n.s	– 0.402 (0.049)

Legend: Spearman Rank correlations (*p* value) after the spinal tap. Ballon steering at enhanced level of difficulty, collecting apples at the lowest level of difficulty

*n.s.*  not significant

## Discussion

NPH is a neurodegenerative disease characterized by progressive gait disturbance, cognitive impairment, urine incontinence and a communicating hydrocephalus as evident from CT or MRI [2, 4]. Its manifestation has recently been shown by dynamic MRI scanning of an intrathecally administered contrast agent to result from an impaired resorption of the cerebrospinal fluid by the glymphatic system [27, 28]. This disturbance has been hypothetized also to cause the intermittent peaks of intracranial pressure that result in periventricular fluid effusion and subsequent degeneration of descending fibre tracts in hemispheric white matter [12, 29].

In this prospective study, we used neuroimaging data and validated quantitative measures of cognitive and gait functions to identify patients with NPH from 47 patients admitted for diagnostic work-up of suspected NPH. We found that there were patients with definite NPH, with possible NPH and patients with neurological abnormalities not compatible with NPH. This accords with the well-known heterogeneity of the clinical presentation of patients assumed to have NPH [7–9]. In addition, we determined by quantitative kinematic measures the arm function in these patients. We found that gait disturbance was associated with compromised control of arm movements. Using rotation movements of the hand we found that in patients with definite NPH the visuomotor control of intermittent movements was severely impaired such that most patients failed at higher levels of difficulty. Although the spinal tap improved this performance in all patients slightly for the right hand, this improvement was significant for the left hand only in the patients with NPH and virtually absent in the two other patient groups. It cannot be excluded that the improvement of the left hand in the patients with NPH may be driven by the two left-handed patients in this patient group. In contrast, the visuomotor control of monotonic, predictable movements was not impaired in the patients with NPH similar to the lack of improvement of the nine-hole-peg test after a spinal tap [30]. We were able to show that gait performance and hand-arm movements were scaled in proportion after the spinal tap as indicated by the non-parametric Spearman rank correlations. Since the patients with NPH showed only a slight elevation of the S100 protein in CSF, the impairment of visuomotor hand coordination was related to the pathophysiology of NPH but not to another type of dementia. For comparison, the patients with no NPH showed unspecific changes of the neurodegenerative markers in CSF and were not impaired in these visuomotor control tasks nor did they improve in gait and cognitive functioning after the spinal tap. The posNPH patients showed deficits and post-tap improvement patterns in between.

From a methodological point of view, it should be pointed out that the data of this study were gathered during the diagnostic work-up of NPH to establish the diagnosis and to predict putative shunt outcome [7, 10]. Importantly, one of the starting points of our study was to assess the cognitive deficits and the impairment of gait in a quantitative fashion to provide observer-independent and reliable measures of the neurological improvement potentially brought about by the spinal tap. We, therefore, adopted dedicated tests that had been validated in the literature [11]. Thereby, we were able to differentiate patients reliably who responded to a spinal tap from those who did not. Patient classification was done by considering the neuropsychological and gait patterns. Given the interrater reliability of the De Morton Mobility Index [20], we adopted a 20% change after the spinal tap to be diagnostic in the individual patient. Furthermore, we used the so-called PABLO^R^-device to determine hand-arm function quantitatively in terms of visuomotor coordination. The PABLO^R^-device is based on digitized accelerometer technology offering quantitative measures of visuomotor hand function as described in detail elsewhere [26]. Moreover, it allowed us to determine the success rate of task performance at different levels of task difficulty.

The patients of the three groups were quite comparable concerning their demographic data (Table 1). It should be pointed out that all had a markedly slowed gait as assessed with the 10MWT, but the patients with NPH were particularly impaired in the TUG test which was significant as compared with the patients with no NPH. Accordingly slowing of gait does not seem to be suited for differentiation of neurological syndromes. The specific aspect of the TUG is the sequence of different motor acts including rising, initiating walking, keep going, and turning around an obstacle [21]. This seems to be comparable to the intermittent motor acts needed to execute the apple collecting task using the PABLO^R^-device. It should be pointed out that this task involved intermittent, unpredictable visuospatial actions. It appears from this study that patients with NPH are particularly impaired to master such composite, higher-order arm movement tasks. Since the supplementary motor area and the pre-supplementary motor area in the frontal midline cortex have been shown by multimodal imaging to mediate differentiated roles in higher-order movement control [31, 32], it is speculated that their damage induces the higher-order control deficits of gait functions and visuomotor arm movements as reported here. The NPH defining hydrocephalus have been shown by MRI to affect the cortico-cortical output of these frontal cortical midline structures that cross to the contralateral hemisphere via the corpus callosum as well as the cortico-subcortical projections that travel in the periventricular vicinity [7, 14].

Importantly, our patients with NPH had the neurodegenerative biomarkers in CSF in normal range and, thus, fulfilled the criteria of idiopathic NPH [7]. This was in contrast to the patients considered not to be compatible with NPH. In these patients with no NPH the astrocytic marker S100 and, in particular, the neuron-specific enolase were elevated probably due to the fact that at least some of these patients suffered from a degenerative brain disease. In contrast, it was recently described that patients with NPH who responded to a spinal tap had abnormal levels of these biomarkers as well as of ß-amyloid, the tau protein and phosho-tau in CSF [32]. It is important to realize, however, that the patients with NPH showing the neurodegenerative biomarkers in CSF were older than the patients with NPH who did not have amyloid, the tau protein and phosho-tau in CSF [33]. The patients in this study were even still younger. Since it was reported recently that ß-amyloid and tau-protein are deposited in cerebral cortex in relation to ageing with the consequence of rapid neurodegeneration and memory decline [34, 35], it can be speculated that at higher age patients with NPH may develop also dementia of neurodegenerative cause. Accordingly, the progressive amyloid and tau accumulation with age and in AD could be due to the failure of the CSF circulation to clear metabolic waste which may occur from decreased CSF production and/or CSF absorption. In line with this assumption, patients with NPH were found to have similar patterns of beta-amyloid and tau in CSF as those with Alzheimer’s disease in a large meta-analysis of 25 studies with over 664 patients [36]. In principle, deposition of beta-amyloid in the meninges may compromise CSF outflow, while abnormalities of CSF production may impair the clearance of beta-amyloid which has been hypothesized to coincide in individual patients [37]. However, from this study it appears that the S100 protein and the neuron-specific enolase are elevated in patients with NPH suggesting that they reflect neurodegeneration before the CSF abnormalities typically for Alzheimer’s disease become manifest. An idiopathic NPH without degenerative CSF changes is supposed to be the appropriate candidate for a ventriculo-peritoneal or ventriculo-caval shunt, even if a sustained improvement upon spinal drainage over three years was reported to be variable [7]. Thus, apart from the increase in mobility the improvement of higher-order visuomotor arm movement control after a spinal tap as described in this study is an important argument in support of shunt installation.

In conclusion, patients with definite NPH were found to exhibit, in addition, to a progressive gait disturbance also an impairment of visuomotor coordination of intermittent arm movements. After the spinal tap this arm motor control deficit was improved in proportion to the improvement of gait suggesting a general motor control abnormality in NPH. It may be speculated that in NPH the periventricular white matter damage affects the nerve fibre tracts originating from the supplementary motor (control) area.

## Data Availability

Original data are available on request to the corresponding author.
